# Results of the CHlorhexidine Gluconate Bathing implementation intervention to improve evidence-based nursing practices for prevention of central line associated bloodstream infections Study (CHanGing BathS): a stepped wedge cluster randomized trial

**DOI:** 10.1186/s13012-021-01112-4

**Published:** 2021-04-26

**Authors:** Staci S. Reynolds, Patricia Woltz, Edward Keating, Janice Neff, Jennifer Elliott, Daniel Hatch, Qing Yang, Bradi B. Granger

**Affiliations:** 1 Duke University School of Nursing, 307 Trent Drive, Durham, NC 27710 USA; 2 Duke University Hospital, 2310 Erwin Road, Durham, NC 27710 USA; 3 WakeMed Health & Hospitals, 3000 New Bern Avenue, Raleigh, NC 27610 USA; 4 Duke University Health System, 2310 Erwin Road, Durham, NC 27710 USA

**Keywords:** Implementation science, Chlorhexidine gluconate, Patient bathing, Nursing care, Catheter-related bloodstream infection, Audit and feedback intervention, Educational outreach

## Abstract

**Background:**

Central line-associated bloodstream infections (CLABSIs) result in approximately 28,000 deaths and approximately $2.3 billion in added costs to the U.S. healthcare system each year, and yet, many of these infections are preventable. At two large health systems in the southeast United States, CLABSIs continue to be an area of opportunity. Despite strong evidence for interventions to prevent CLABSI and reduce associated patient harm, such as use of chlorhexidine gluconate (CHG) bathing, the adoption of these interventions in practice is poor.

The primary objective of this study was to assess the effect of a tailored, multifaceted implementation program on nursing staff’s compliance with the CHG bathing process and electronic health record (EHR) documentation in critically ill patients. The secondary objectives were to examine the (1) moderating effect of unit characteristics and cultural context, (2) intervention effect on nursing staff’s knowledge and perceptions of CHG bathing, and (3) intervention effect on CLABSI rates.

**Methods:**

A stepped wedged cluster-randomized design was used with units clustered into 4 sequences; each sequence consecutively began the intervention over the course of 4 months. The Grol and Wensing Model of Implementation helped guide selection of the implementation strategies, which included educational outreach visits and audit and feedback. Compliance with the appropriate CHG bathing process and daily CHG bathing documentation were assessed. Outcomes were assessed 12 months after the intervention to assess for sustainability.

**Results:**

Among the 14 clinical units participating, 8 were in a university hospital setting and 6 were in community hospital settings. CHG bathing process compliance and nursing staff’s knowledge and perceptions of CHG bathing significantly improved after the intervention (*p* = .009, *p* = .002, and *p* = .01, respectively). CHG bathing documentation compliance and CLABSI rates did not significantly improve; however, there was a clinically significant 27.4% decrease in CLABSI rates.

**Conclusions:**

Using educational outreach visits and audit and feedback implementation strategies can improve adoption of evidence-based CHG bathing practices.

**Trial registration:**

ClinicalTrials.gov, NCT03898115, Registered 28 March 2019.

**Supplementary Information:**

The online version contains supplementary material available at 10.1186/s13012-021-01112-4.

Contributions to the literature

- Daily chlorhexidine gluconate (CHG) bathing to decrease patients’ risk of infection is widely supported in the literature. However, adoption of this practice varies greatly.
- An implementation science process model, Grol and Wensing’s Model of Implementation, guided identification of barriers and selection of implementation strategies.
- Evidence-based implementation strategies of educational outreach visits and audit and feedback were used to improve compliance with daily CHG bathing processes, which were sustained at 12 months.
- These findings contribute to the recognized gap between research and practice and describe effective strategies to implement evidence at the bedside.

## Background

Central line-associated bloodstream infections (CLABSIs) are one of the most common health care-associated infections, accounting for approximately 80,000 infections and up to 28,000 deaths in the United States (U.S.) annually [1–3]. Additionally, CLABSIs contribute $46,000–$75,000 per infection in added costs to the U.S. healthcare system, yet these infections are largely preventable when evidence-based guidelines are consistently incorporated into patient care [4–6]. As a major quality indicator for hospitals, CLABSIs contribute to increased length of stay, morbidity, mortality, and unnecessary antibiotic use [1]. To decrease hospital-acquired CLABSIs, preventive interventions are recommended, such as line insertion and maintenance bundles [7].

One intervention that has been studied extensively is daily bathing of hospitalized patients with 2% chlorhexidine gluconate (CHG) [8–11]. The Centers for Disease Control and Prevention and the Agency for Healthcare Research and Quality (AHRQ) have strongly recommended its use in the intensive care and bone marrow transplant settings to reduce CLABSIs [12]. In 2013, the AHRQ published a protocol for how to effectively use pre-packaged CHG cloths to optimize infection reduction, including (1) bathing patients daily from their jawline to their toes, (2) cleaning over transparent central line dressings and down six inches of the tubing, and (3) cleaning around the perineal area and down 6 in of the indwelling urinary catheter [12]. Daily CHG bathing is widely supported in the literature, with studies showing significant decreases in CLABSIs when CHG bathing is performed correctly and consistently [9–11, 13, 14]. Despite strong evidence for CHG bathing to prevent CLABSIs, the adoption of this intervention in practice per the AHRQ protocol is poor [15–18].

Previous studies have found that daily CHG bathing is only completed 23–77% of the time [16–19]. Reynolds and colleagues [17] noted that, although nurses had been educated on using CHG bathing daily, they were unaware of the AHRQ protocol and the appropriate procedure for bathing, including cleaning over transparent central line dressings and six inches of the tubing. Other barriers noted in the literature include a lack of time, lack of motivation, and a lack of perceived importance of CHG bathing in reducing infections [16–18]. A recent mathematical modeling and cost analysis study [20] showed that increasing CHG bathing compliance from 60 to 90% had the potential to reduce CLABSI incidence by 32%, result in 20 averted infections, save over $815,000, and decrease mortality—saving up to 5 lives. Due to poor adoption of evidence-based CHG bathing practices, nurses are missing opportunities to reduce infection rates in critically ill patients. As such, there is a great need to better implement this seemingly simple intervention into practice.

A long-standing question in health care is how to implement evidence into practice in an efficient, equitable, timely and patient-centered way. In 2001, the National Academy of Medicine (formerly Institute of Medicine) report, “Crossing the Quality Chasm: A New Health System for the 21st Century” brought national attention to major, systematic barriers for delivering high quality of care [21]. This quality gap translated into an average of 17 years for new knowledge generated by randomized controlled trials to be incorporated into practice, and even when done, frequently with disparities. Unfortunately, this gap in translating evidence into practice remains with respect to CLABSI prevention, due in part to a lack of evidence on best implementation strategies. Though strategies for implementation have been well-studied in other contexts and populations, with use of educational outreach visits and audit and feedback being the most effective [22], these strategies have not been widely reported in the setting of CHG bathing interventions [17, 23, 24].

To meet the values outlined by the National Academy of Medicine almost two decades ago and close the evidence-into-practice gap, a need exists to develop, evaluate, and implement solutions that more effectively drive evidence into practice. The purpose of this study was to address CLABSI rates that were higher than the targeted average at two large health systems in the southeast U.S. We sought to identify effective, tailored strategies to improve adoption of evidence-based bathing practices, and to implement CHG bathing effectively and consistently among nursing staff using the implementation strategies of (1) educational outreach visits and (2) audit and feedback.

The primary objective was to assess the effect of this implementation strategies program on nursing staff’s compliance with the CHG bathing process and electronic health record (EHR) documentation. Secondary objectives were to examine the (1) moderating effect of unit characteristics and cultural context on the intervention, (2) intervention effect on nursing staff’s knowledge and perceptions of CHG bathing, and (3) the intervention effect on CLABSI rates. Primary outcome measures were assessed 12 months after the intervention to assess for sustainability.

## Methods

### Study design

We conducted a multicenter, pragmatic cluster randomized, stepped wedged cross-sectional study to evaluate the effect of using a tailored implementation strategies program on nursing staff compliance with daily CHG bathing processes and documentation per the AHRQ protocol for critically ill patients. A cluster randomized design was chosen with hospital units clustered for implementing the intervention to minimize risk of cross contamination during the study. A stepped wedge design was chosen so all hospital units could sequentially implement the intervention. Eligible hospital units were strategically grouped into four clusters to ensure all sites were represented in each cluster and the opportunity for events (i.e., central venous catheter [central line] device days) was balanced. These four clusters were then randomized to four sequences to start study interventions through a random number generator completed by members of the study team. The study spanned the course of 5 months, with the evidence-based implementation strategies program (the intervention) carried out over 4 months from June to September 2019; May 2019 served as a baseline month (Table 1). Twelve months after the initial intervention (September 2020) booster sessions, which included use of educational outreach visits and audit and feedback strategies, were implemented to assess sustainability. During this month, process and documentation audits were conducted to assess compliance. The study was reviewed by both Duke University and WakeMed’s Institutional Review Boards and determined to be exempt, meaning no more than minimal risk and not meeting the definition of human subject research as defined by federal regulation 45 CFR 46 [25].

**Table 1:**
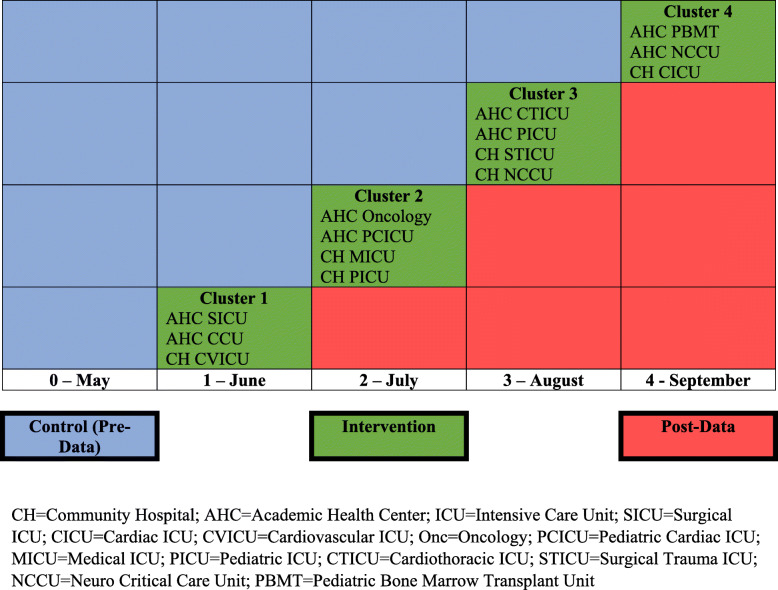
Stepped wedge sequencing of unit clusters

*CH* community hospital, *AHC* academic health center, *ICU* intensive care unit, *SICU* surgical ICU, *CICU* cardiac ICU, *CVICU* cardiovascular ICU, *Onc* oncology, *PCICU* pediatric cardiac ICU, *MICU* medical ICU, *PICU* pediatric ICU, *CTICU* cardiothoracic ICU, *STICU* surgical trauma ICU, *NCCU* neuro critical care unit, *PBMT* pediatric bone marrow transplant unit

### Setting and participants

The study was conducted in two large hospitals in the southeastern U.S.: one academic (957 total beds) and one community-based (683 total beds). Units were included in the study if they admitted critically ill patients and had at least 1 CLABSI event over the past 12 months. The 14 units included in this study ranged in size from 8 to 32 beds and included 9 adult intensive care unit (ICUs), 3 pediatric ICUs, one pediatric bone marrow transplant unit, and one adult hematology/oncology unit (See Supplemental Table 1). Prior to starting the study, monthly unit central line days ranged from 10 to 900. The end users of the practice change were the nursing staff (registered nurses [RN] and nursing assistants [NA]) who worked on the units and provided patient baths.

### Processes

An implementation science process model, the Grol and Wensing Model of Implementation, was used to guide the study [26] (see Table 2). This process model was chosen as it can assist in implementation by offering practical guidance in planning, strategy selection, and execution of implementation studies. Per the model, the first step is to analyze current performance [26]. Analysis of actual performance at both hospitals showed that CHG baths were not consistently documented daily and that RNs and NAs were not following the AHRQ protocol for bathing. For example, staff often failed to use CHG cloths to clean over transparent central line dressings or down catheter tubing. Upon discussion with nursing staff, it was found that they were unaware of the AHRQ protocol and necessary components of the CHG bathing process. Also, nurses noted that the task of patient bathing was given a low priority compared to other care requirements; because of this low priority, nurses were not provided feedback with how well they were doing with bathing. These barriers, including a lack of knowledge, awareness, and perceived importance, helped guide selection of implementation strategies. The Grol and Wensing Model of Implementation indicates that implementation strategies may be more effective if they are tailored to the identified barriers [26]. The implementation strategies of educational outreach visits and audit and feedback were chosen based on the identified barriers; strategies are described in detail below.

**Table 2:** Grol and Wensing model of implementation

Step 1	Step 2	Step 3	Step 4	Step 5
Develop a proposal for change	Analyze actual performance, target group and setting	Development and selection of strategies and measures to change practice	Development, testing, and execution of implementation plan	Continuous evaluation and (where necessary) adapting plan
Development of local guidelines based off of AHRQ resources; specific, concise, and relevant educational flyers developed from these resources for nursing staff	Baseline measurement of process and documentation compliance. Survey of staff to understand their knowledge of CHG bathing. Informal discussions with staff to identify barriers and facilitators of guideline use.	Linked identified barriers (lack of knowledge, and perceived importance) to implementation strategies	Planning, preparation, and execution of implementation strategies (educational outreach visits and audit and feedback)	Follow up measurement (post-data) and sustainability plan

### Educational outreach visits

O’Brien and colleagues [27] describe educational outreach visits where trained professional experts, either internal or external to the environment, visit clinicians in their practice areas and provide them with information on how to change and improve their practice through research-based evidence. The goal of using educational outreach visits was aimed at decreasing the knowledge deficit and improving the priority given to CHG bathing by nursing staff. In our study, experts included infection prevention staff, clinical nurse specialists, and trained members of the research team who were all internal to each organization, but external to the local units. The research team developed a script to ensure consistent messaging for the outreach visits. Members of the research team met with unit leadership prior to their intervention month to identify the best times and venues for disseminating this information on each unit. The face-to-face educational outreach visits took place in staff meetings, unit huddles, and/or informal rounding on the unit and lasted approximately 5–15 min. The experts visited each unit 1–4 times a week on various shifts to reach as many staff as possible during the unit’s intervention month.

During the visits, experts educated nursing staff on (1) the current gap in practice, (2) the rationale for how CHG can decrease CLABSIs and why CHG baths are important, and (3) the proper process for bathing patients with CHG cloths per the AHRQ protocol. A key component during these visits was to increase the priority given to the bath by messaging CHG bathing as not “just a bath” but rather a topical “antimicrobial treatment” administered to decrease risk of infection. With each interaction, the educational outreach visit was personalized to the knowledge base of individual staff members. Questions from staff were encouraged and barriers to CHG bathing were discussed. Barriers were addressed by the research team. Educational materials provided during the visits included a CHG Bathing Frequently Asked Questions sheet and a CHG bathing resource packet. Educational outreach visits were performed for each cluster of units during their intervention month.

### Audit and feedback

According to Ivers and colleagues [28], audit and feedback is a strategy based on the belief that clinicians are prompted to change their behavior and practice when routinely provided feedback on their performance. With this underpinning belief, the goal of using audit and feedback for our study was to provide nursing staff with an awareness of their compliance with CHG bathing.

Nursing staff were provided audit and feedback data regarding their compliance with (1) the CHG bathing process per the AHRQ protocol, and (2) their documentation of daily CHG bathing in the EHR. Once units were enrolled into their intervention month, audit data were provided to units weekly. Data were either posted on the unit and/or sent to nursing staff and unit leadership via email. The method of audit and feedback delivery was determined by the research team in collaboration with the local unit; in implementation science, it is important and necessary to modify strategies to fit the established processes at the local level. Included in the emails and unit postings were (1) run charts of compliance (documentation and process) to allow nursing staff to see their unit’s progress visually and in writing, (2) along with a “kudos” section that listed the names of RNs and NAs who appropriately documented in the audited charts. In addition to sharing documentation and process compliance, units were also provided monthly CLABSI data.

### Booster sessions

In September 2020 (12 months following the initial intervention), booster sessions were conducted in all 14 participating units. During the booster sessions, educational outreach visits and audit and feedback strategies were used in the same manner as in the initial implementation. Process and documentation compliance, as well as CLABSIs rates, were monitored during this month as well. As in the initial implementation intervention, audit and feedback data were provided to the units on a weekly basis during September 2020.

### Measures

#### Primary outcomes

##### CHG bathing process compliance

Per the AHRQ protocol, the entire body below the jawline should be bathed with CHG, including over the transparent central line dressing and down the first 6 in of the catheter/extension tubing as well as around the perineal area and down 6 in of the indwelling urinary catheter tubing. Infection prevention champion nurses who worked on the units completed process audits through observation of nursing staff providing CHG baths. Each unit had 2–5 champions who were coached and then provided with an instructional video on how to complete the audit. Champions recorded how many appropriate body parts were bathed with CHG cloths (see Table 3). Since it is difficult to remain anonymous while observing a patient being bathed in a private setting, champions remained anonymous to the extent possible and attempted to conduct process audits without staff awareness. Champions were instructed to physically observe a bath being performed by a fellow RN or NA. However, if unable to complete an observation, champions were instructed to retrieve data via a self-reported audit from the staff who bathed the patient. Data was entered into REDCap© which calculated a compliance percentage. Bathing process audits were completed by champions 4 to 8 times per month. The compliance rate is calculated for each month.

**Table 3:** Process audit form

Question	Yes	No	N/A	Comments
CHG cloth used for neck, shoulders, and chest				
Applied CHG over central line(s) dressing(s)				
Cleaned the closest 6′′ of line(s) with CHG cloth				
6.1.1.1.1. CHG cloth used for both arms, hands, and armpits				
Applied CHG over PICC/line(s) dressing(s)				
Cleaned the closest 6′′ of PICC/PIV lines with CHG cloth				
6.1.1.1.2. CHG cloths used for abdomen, groin, and perineum				
Clean around indwelling Foley catheter and down 6′′ of the tubing				
6.1.1.1.3. CHG cloths used for right leg and foot				
6.1.1.1.4. CHG cloths used for left leg and foot				
6.1.1.1.5. CHG cloths used for back of neck, back, and buttocks				

##### CHG bathing EHR documentation compliance

Throughout the study, the EHR for each unit was audited every week to determine compliance with CHG bathing documentation. Once a week, charts from all patients with central lines were audited to see whether a “CHG Bath” was documented within the previous 24-h period. Two members of the research team conducted audits and entered their findings into REDCap©. Patient records were deemed ineligible and excluded if patients had a CHG allergy or had been admitted to the unit for less than 24 h. Compliance with CHG bathing documentation was measured as the number of eligible patients who had a CHG bath documented divided by the total number of eligible patients. The compliance rate is calculated for each month.

#### Moderators

##### Hospital unit characteristics

Several hospital unit characteristics were included as moderators in this study; see Table 4 and supplemental table 1.

**Table 4:** Cluster characteristics

Characteristic	A	B	C	D	*p* value
# of beds	20.33 (3.51)	16.75 (10.14)	16.25 (11.09)	19.33 (4.16)	.60
CL utilization	2.74 (1.96)	3.66 (3.68)	2.61 (1.22)	2.53 (1.27)	.99
RN hours ppd	21.64 (0.67)	19.95 (7.02)	23.78 (3.88)	19.87 (1.45)	.45
# RN FTE	60.87 (27.43)	47.65 (26.36)	65.09 (61.28)	55.87 (34.05)	.89
Staff Turnover	28.21% (11.47)	12.18% (11.09)	20.52% (11.15)	19.23% (5.36)	.40
Skill Mix	92.37% (5.82)	85.63% (7.21)	90.18% (5.23)	88.07% (5.59)	.52
# of admissions per month	141.00 (23.58)	77.75 (19.62)	106.00 (47.25)	115.33 (80.98)	.34
LOS	3.67 (1.01)	6.37 (4.70)	3.41 (0.68)	7.16 (6.63)	.95
Total years of RN experience	7.35 (3.16)	9.77 (3.46)	6.83 (2.16)	9.26 (5.30)	.55
Total years of NA experience	8.13 (6.31)	7.84 (3.89)	10.59 (5.34)	9.03 (5.55)	.78
Average RN age	32.28 (4.79)	34.97 (4.75)	32.04 (3.20)	34.55 (6.61)	.82
Average NA age	39.61 (5.53)	33.40 (4.58)	35.21 (6.88)	39.42 (8.42)	.55

*A* group randomized in June, *B* group randomized in July, *C* group randomized in August, *D* group randomized in September. Skill mix = RN nursing care hours as a % of all nursing care hours. Kruskall-Wallis tests conducted

*CL* central line; *ppd* per patient day, *FTE* full time employee, *LOS* length of stay

##### Context Assessment Index

For successful implementation, evidence needs to be robust, the context receptive to change, and the change appropriately facilitated [29]. The Context Assessment Index (CAI) is an instrument to evaluate the context of practice and its readiness to implement evidence into practice. The CAI is a self-report instrument consisting of 37 items with 4 responses on a Likert scale of strongly agree to strongly disagree. The scoring tool yields an overall score and 3 sub-scores for leadership, culture, and evaluation. High values indicate a high/strong context. Previously, the CAI demonstrated acceptable psychometric properties with Cronbach’s alpha estimated at 0.93 for the total score [30].

The CAI was sent to infection prevention champions in May 2019 via REDCap© prior to the beginning of the intervention. Nine demographic questions were also sent. The CAI was administered to assess the context (i.e., culture) in which clinicians worked to evaluate the effect it might have on using evidence in practice; this survey was considered a moderator for the analysis, along with other unit characteristics. The CAI for each unit was calculated by averaging among all the champions if available. Consent was implied if the staff member clicked on the REDCap© link and participated in the survey.

#### Secondary outcomes

##### Knowledge/perceptions of CHG bathing surveys

A knowledge and perceptions survey, adapted from Hines et al. [16], was administer in May 2019 via REDCap© to all nursing staff on each unit. A post-survey was automatically sent in October 2019 to RNs and NAs who completed the pre-survey. The anonymous survey included 12 demographic questions and 12 knowledge and perception questions. The goal of this survey was to assess staff’s knowledge of CHG bathing, as well as their perceptions of the importance and priority given to CHG bathing. Consent was implied by the staff members who clicked on the REDCap© link and participate in the survey.

##### CLABSI rates

CLABSI rates for each hospital unit were captured by the hospitals’ Infection Prevention departments based on standard National Healthcare Safety Network criteria and reported monthly [31]. Data were then entered monthly into the REDCap© database.

### Statistical analysis

Characteristics of each hospital unit and by randomization groups are provided (Table 4 and supplemental table 1). To assess the effect of the implementation strategies on nursing staff compliance with the CHG bathing process and EHR documentation, we first illustrate change in outcomes by using empirical summary plots with means and standard deviations of each of the outcomes by randomization group over the implementation period (May 2019 to October 2019). We then use generalized linear mixed models (GLMM) applicable to testing for intervention effects in cross-sectional stepped wedge designs [32]. In these models, compliance with CHG bathing processes and EHR documentation in the various hospital units over this time period is regressed on the intervention, defined as 0 before implementation and 1 after. To account for clustering within hospital units, a random intercept is included. Because entry into intervention is staggered in stepped wedge designs, the effect of the intervention is confounded with time. To account for this, and to capture the time trend, the fixed effect quadratic effect of time was first tested, followed by the linear effect of time if quadratic time was not significant. In models of compliance with CHG bathing process, observation type was also entered in a separate model. To assess the effect of the follow-up booster session on compliance with CHG bathing process and EHR documentation, an additional GLMM model was tested to compare compliance in September 2019 with compliance in September 2020.

To assess the impact of hospital unit context and other hospital unit characteristics on the effect of implementation strategies in changing compliance, GLMM models of time and intervention were extended to include interactions between intervention and Context Assessment Index (CAI) scores, centered, and between intervention and other hospital unit characteristics. Each of these moderators were tested in separate models.

Demographic characteristics of nursing staff who participated in the knowledge and perception survey are summarized by randomization cluster, using means and standard deviations for continuous variables and frequencies and percentages for categorical variables. Equivalence among randomization clusters was tested using ANOVA for continuous variables and Chi square tests for categorical variables. To assess the impact of the implementation strategies on change in nursing staffs’ knowledge and perception of CHG bathing, McNemar’s chi-square tests were used for categorical variables and paired ***t*** tests for continuous variables.

To assess the intervention effect on unit CLABSI rates, we calculated the average CLABSI rate over the implementation period (May 2019 to October 2019) across all hospital units before and after the intervention. Linear regression models were used to test the intervention effect, in which CLABSI rates in each unit during this time were regressed on time in months and intervention. In these models, the quadratic effect of time was first tested, followed by the linear effect of time. To assess the effect of the follow-up booster session on CLABSI rates, an additional linear regression model was used to examine change in CLABSI rates from October 2019 to September 2020.

## Results

Cluster characteristics are reported in Table 4. Mean number of beds in each cluster varied, from 16.25 to 20.33, as did mean number of admissions, from 77.75 to 141.0. Percent staff turnover ranged from 12.18% to as high as 28.21%. Skill mix was high (range 85.63% to 92.37%). Mean central line utilization ranged from 2.61 to 3.66, and mean RN hours per patient day ranged from 19.87 to 23.78. Number of RN full time employee (FTE) varied widely (range 47.65 to 65.09), as did length of stay (range 3.41 to 7.16).

### CHG bathing process and EHR documentation compliance

**Fig. 1:**
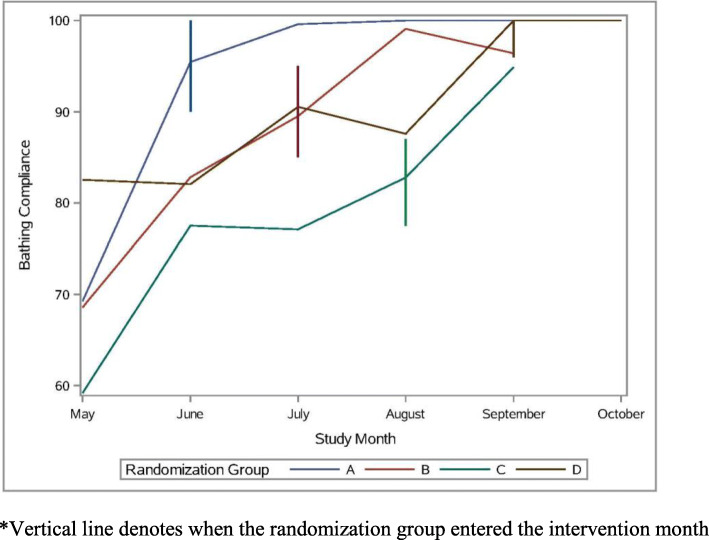
CHG Bathing process compliance by month. *Vertical line denotes when the randomization group entered the intervention month

**Fig. 2:**
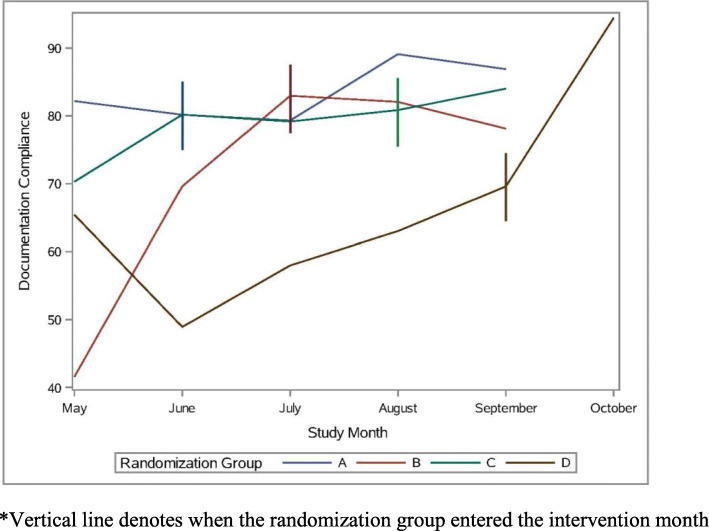
CHG bathing documentation compliance by month. *Vertical line denotes when the randomization group entered the intervention month

A total of 424 process audits were completed; 294 observed and 130 self-reported process audits. Table 5 reports results of GLMM models regressing nursing staff compliance with the CHG bathing process and EHR documentation on implementation strategies and quadratic time in calendar months. In models of CHG bathing process compliance, the implementation strategy was significant (*b* = 6.97, *p* = .009), indicating that compliance was 6.97% higher after the intervention than before. The main effect of time in months and quadratic time were significant (*b* = 9.92, *p* < .001; *b* = − 1.39, *p* = .002), indicating a 9.92 increase in compliance per month at the beginning of the implementation period and a 1.39 decrease in this effect per month. This is consistent with the empirical summary plot of this outcome (see Fig. 1), which shows a tapering of the effect over time. A follow up model indicated that observation type was not significant (*b* = − 3.69, *p* = .06). An additional follow-up model to test the booster session revealed no change in bathing process compliance after 12 months (*b* = − 0.19, *p* = .87, intercept = 96.96, *p* < .001), indicating that compliance remained high at 96.96%.With regard to EHR documentation compliance, a total of 298 documentation audits were performed over the course of the study. In models of EHR documentation compliance, the intervention effect was 6.81%, but was not statistically significant (*b* = 6.81, *p* = .15; see Fig. 2). In this model, quadratic time was not significant (*b* = − 0.54, *p* = .46), and a follow-up model revealed that linear time was also not significant (*b* = 2.36, *p* = .12). An additional follow-up model to test the booster session revealed no change in documentation compliance after 12 months (*b* = 3.89, *p* = .37, intercept = 78.72, *p* < .001), indicating that compliance remained high at 78.72%.

**Table 5:** CHG bathing process and documentation compliance and CLABSI rates on implementation strategies and quadratic time

	Process compliance	Documentation compliance	CLABSI rates
	Estimate (*p* value)	Estimate (*p* value)	Estimate (*p* value)
6.1.1.1.6. intercept	60.40, *p* < .001	59.55, *p* < .001	*b* = 4.23, *p* = .004
6.1.1.1.7. Time (months)	*b* = 12.70, *p* < .001	*b* = 5.67, *p* = .24	*b* = -1.24, *p* = .43
6.1.1.1.8. Time * time	*b* = -1.39, *p* = .002	*b* = -0.54, *p* = .46	*b* = 0.05, *p* = .84
6.1.1.1.9. intervention	*b* = 6.97, *p* = .009	*b* = 6.81, *p* = .15	*b* = 1.22, *p* = .56

### Moderating effect of context and unit characteristics

Results of GLMM models regressing nursing staff compliance with CHG bathing process and EHR documentation on implementation strategies moderated by CAI and hospital unit characteristics are reported in Table 6. A total of 30 champions completed the CAI. Findings for bathing process compliance revealed a significant intervention by CAI score interaction (*b* = − 0.90, *p* < .001). This indicates that each one-point increase in CAI total score is associated with a − 0.90 decrease in the effect of the intervention on process compliance. The interaction between intervention and number of beds was also significant (*b* = − 0.77, *p* = .002), indicating that every one unit increase in the number of beds is associated with a − 0.77 decrease in the effect of the intervention on this outcome. The interaction was also significant for RN hours per patient day (ppd) (*b* = 1.32, *p* = .004), with every additional RN hour ppd being associated with a 1.32 increase in the effect of the intervention. Length of stay also significantly interacted with intervention (*b* = − 1.67, *p* < .001), with every 1 day increase in length of stay being associated with a 1.67 decrease in the intervention effect. For this outcome, interactions for all other unit characteristics were non-significant.

**Table 6:** Moderating effect of context and other unit characteristics on CHG bathing process and documentation compliance

Interaction between intervention	Process compliance		Documentation compliance	
	Estimate	*p* value	Estimate	*p* value
CAI	− 0.90	< .001	0.98	.03
# of beds	− 0.77	.002	− 0.41	.29
CL utilization	0.64	.44	− 0.14	.93
RN hours ppd	1.32	.004	0.36	.58
# RN FTE	− 0.10	.05	− 0.07	.40
Staff Turnover	− 0.04	.82	0.10	.72
Skill Mix: RN Nursing care hours as a % of all nursing care hours	0.08	.81	− 0.13	.79
# of admissions per month	0.03	.54	− 0.14	.02
LOS	− 1.67	< .001	1.17	.13
Total years of RN experience	1.00	.08	0.78	.36
Total years of NA experience	0.60	.09	− 0.95	.13
Average RN age	0.71	.09	0.35	.59
Average NA age	0.37	.19	− 1.01	.02

For EHR documentation compliance, the intervention by CAI score interaction was significant (*b* = 0.98, *p* = .03), indicating each one-point increase in CAI score to be associated with a 0.98 increase in the effect of the intervention on EHR documentation compliance. The interaction between intervention and number of admissions per month was also significant for this outcome (*b* = − 0.14, *p* = .02), indicating each additional admission to be associated with a − 0.14 decrease in the intervention effect for this outcome. The interaction was also significant for average NA age (*b* = − 1.01, *p* = .02), with each one year associated with a − 1.01 decrease in the intervention effect. For this outcome, interactions for all other unit characteristics were non-significant.

### Knowledge/perceptions of CHG bathing surveys

Characteristics of survey participants by randomization group are reported in Table 7. Mean age among all participants was 32.21 (SD = 9.52). There was a statistically significant difference in mean age across different randomization groups (*F* = 5.5, *p* = .001); however, the biggest difference in mean age is 5.58 (between group A [M = 29.45, SD = 6.67] and group C [M = 35.03, SD = 9.89]), which is not clinically significant. Most participants were female (87.46%), non-Hispanic (96.55%), and White (80.76%). Mean years of experience was 7.36 (SD = 8.78), though this also differed across randomization groups (*F* = 4.48, *p* = .004).

**Table 7:** Demographics of nursing staff who completed the knowledge/perceptions survey by randomization group

	All participants *n* = 325	Group A *n* = 72	Group B *n* = 73	Group C *n* = 110	Group D *n* = 69	Test value	*p*-value
Age	32.21 (9.52)	29.45 (6.67)_a_	35.03 (9.89)_b_	30.92 (9.17)_a_	34.13 (11.09)_b_	*F* = 5.50	.001
Gender: Female	279 (87.46%)	61 (88.41%)	64 (91.43%)	95 (87.96%)	58 (92.06%)	χ^2^ = 1.08	.78
Ethnicity: Hispanic	11 (3.45%)	3 (4.76%)	2 (3.03%)	3 (2.94%)	3 (5.08%)		.86
Race							.41
Asian	4 (1.26%)	0 (0%)	0 (0%)	4 (3.96%)	0 (0%)		
Black	22 (6.94%)	4 (6.67%)	3 (4.62%)	9 (8.91%)	6 (10.0%)		
White	256 (80.76%)	55 (91.67%)	61 (93.85%)	86 (85.15%)	53 (88.33%)		
More than one race	5 (1.58%)	1 (1.67%)	1 (1.54%)	2 (1.98%)	1 (1.67%)		
Years of nursing experience	7.36 (8.78)	4.99 (6.17)_a_	8.88 (8.91)_bc_	6.36 (7.83)_ab_	9.73 (11.28)_c_	*F* = 4.48	.004

Means with different subscripts statistically significant. Fisher’s exact test conducted for Ethnicity and Race

*A* group randomized in June, *B* group randomized in July, *C* group randomized in August, *D* group randomized in September

Results of analyses examining change in these participants’ knowledge and perception of CHG bathing is summarized in Table 8. Of the 325 survey participants, 90 completed both the pre and post surveys. Findings revealed that the percentage of participants correctly identifying facts about CHG bathing increased, from 31.11 to 50.0% (χ^2^ = 9.32, *p* = .002). In addition, mean perception of the priority of CHG bathing increased, from pre-survey (*M* = 2.60, SD = 0.79) to post-survey (*M* = 2.79, SD = 0.75) and this difference is statistically significant (*t* = 2.56, *p* = .01). Other aspects of knowledge and perception did not change.

**Table 8:** Change in knowledge and perception from pre to post, *n* = 90

	Pre	Post	Test statistic	*p* value
Knowledge				
Reasons for using CHG: correct	32 (35.56%)	32 (35.56%)	χ^2^ = 0	1.0
Facts about CHG: correct	28 (31.11%)	45 (50.0%)	χ^2^ = 9.32	.002
CHG decreases CLABSI	3.11 (0.76)	3.24 (0.84)	*t* = 1.27	.21
Perception				
Importance of bathing	3.30 (0.73)	3.28 (0.78)	*t* = − 0.33	.74
Priority of bathing	2.68 (0.77)	2.74 (0.77)	*t* = 0.82	.41
Importance of CHG bathing	2.81 (0.93)	2.93 (1.01)	*t* = 1.37	.17
Priority of CHG bathing	2.60 (0.79)	2.79 (0.75)	*t* = 2.56	.01

For “Knowledge: reasons for using CHG” and “Knowledge: facts about CHG,” *n*-size and percent correct are reported, and McNemar’s chi-square tests are reported to assess change. For remaining items, means and standard deviations are reported, and paired *t* tests are used to assess change

### CLABSI rates

**Fig. 3:**
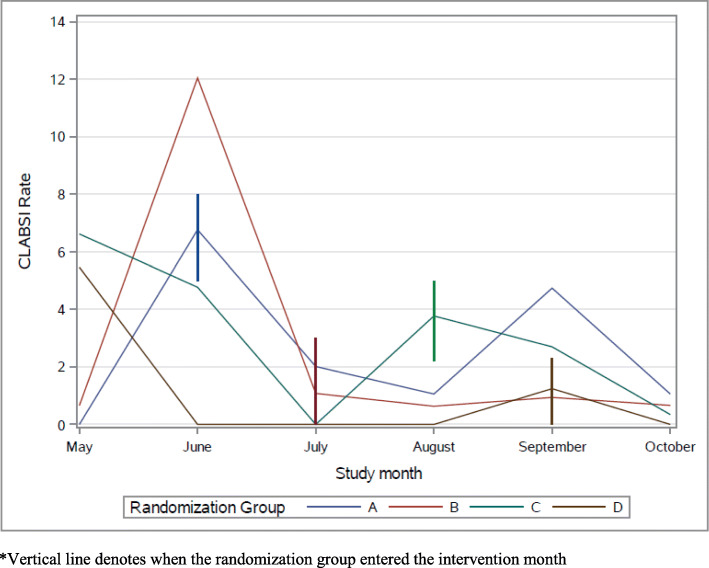
CLABSI Rates by month. *Vertical line denotes when the randomization group entered the intervention month

**Fig. 4:**
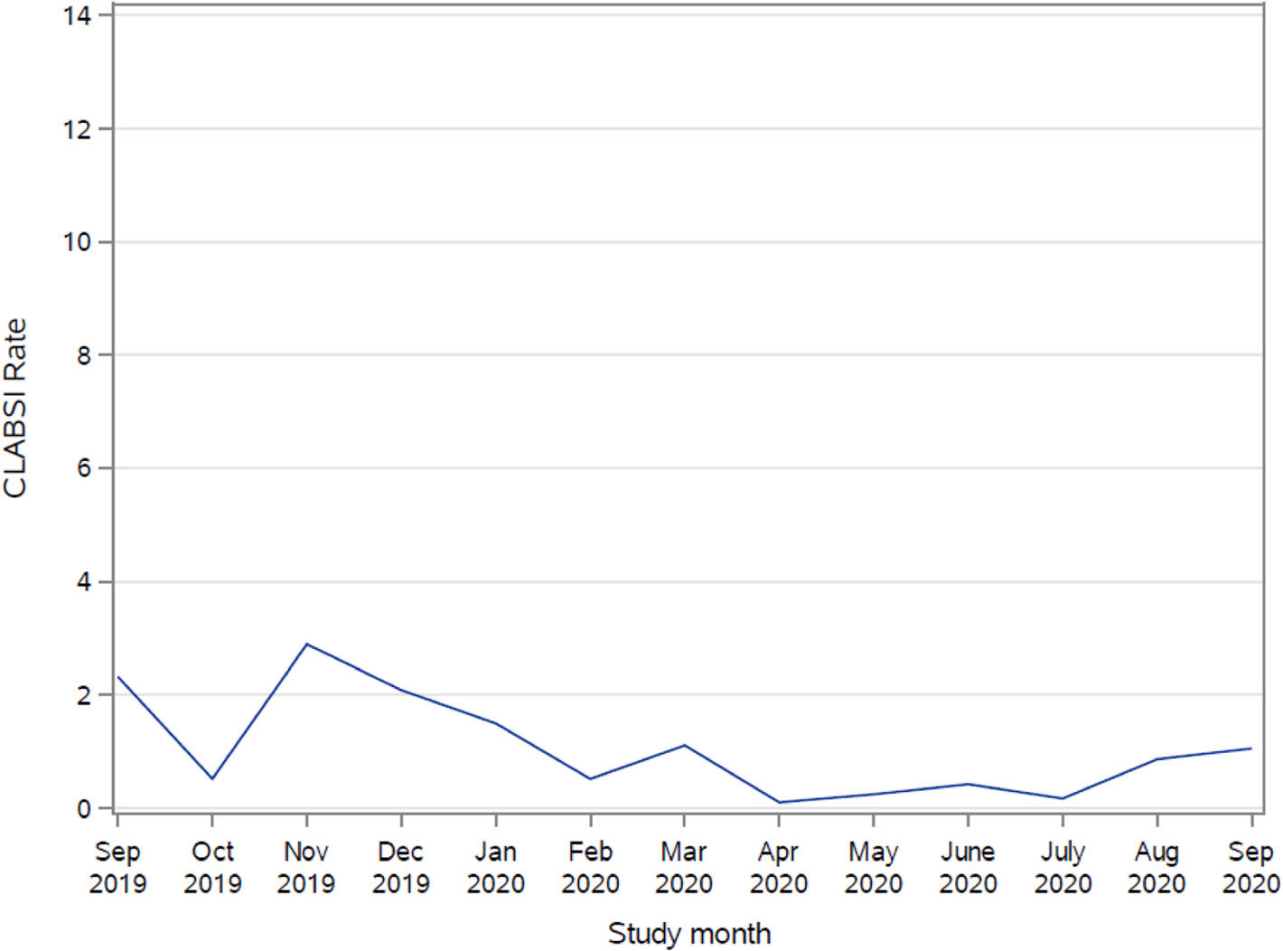
CLABSI rates over follow-up period

Although the study was not powered to look at the effect of the intervention on CLABSI rate, actual rates of CLABSI decreased 27.4% (from 2.59 to 1.88) on average from baseline to post intervention. Linear regression models examining the effect of implementation strategies on unit CLABSI rates are presented in Table 5. Findings revealed the effect of quadratic time to be non-significant (*b* = 0.05, *p* = .84; see Fig. 3), and the intervention effect to be non-significant (*b* = 1.22, *p* = .56). The effect of linear time was also non-significant (*b* = − 0.96, *p* = .11). An additional follow-up model to test the booster session revealed a decrease in CLABSI rates over the 12-month follow-up period (*b* = − 0.16, *p* = .009, intercept = 1.97, *p* < .001; see Fig. 4), indicating that at 12 months post-intervention, CLABSI rates were 1.81.

## Discussion

The major finding of this cluster randomized, stepped wedge controlled trial for CHG bathing demonstrates the ability of an implementation program intervention to effectively change routine nurse behaviors, resulting in improved adoption of guideline-based CHG bathing processes, improved documentation of bathing care delivery, improved knowledge and perceptions of CHG bathing among nursing staff, and a clinically meaningful 27.4% reduction in CLABSI rates. The study is unique in that the strategies used for delivery of the intervention, that is, educational outreach visits together with audit and feedback, served to remind, reinforce, and lend authoritative value to the CHG bathing intervention throughout the implementation process. These strategies not only improved unit bathing processes during the period of the intervention, but in some units also demonstrated improved care delivery processes for up to three months beyond the immediate intervention.

Stepped wedge cluster randomized trial design is a novel design that is increasingly used in implementation science. It offers great flexibility to carry out a pragmatic study trial. It also minimizes the confounding as each cluster contributes to both exposed and unexposed observations. There may be some temporal confounding due to the fact that different clusters received the intervention at different times, but we adjusted for this in the analysis stage by including time as a covariate [33].

### Audit and feedback and educational outreach implementation strategies

Findings of this study support others in the literature that demonstrate the effectiveness of these two implementation strategies, educational outreach visits and audit and feedback, in changing behaviors associated with health care delivery in practice settings. In this study, like that published by Chan and colleagues [22], the timing and persistence of the change over time was strongly correlated with the timing, frequency, and intensity of feedback given. Previous studies have shown poor adoption of CHG bathing, which may be due to poor quality system [16–19]. As demonstrated in our study, educational outreach visits and audit and feedback strategies can remediate these care delivery systems and improve the quality of care provided to patients.

Our study differs from others in two main ways; first, in how strategies were determined and secondly, in how compliance was measured. Many previous studies have sought to improve compliance with CHG bathing to decrease CLABSI rates [18, 34, 35]. Reynolds and colleague s[17] implemented CHG bathing in a neurosurgical ICU setting through using educational outreach visits, audit and feedback, printed educational materials, and local opinion leaders. This article provided details on how strategies were identified and details for how they were operationalized [17]. However, many other articles do not tailor strategies to determinants, and lack the details needed to replicate the implementation strategies [18, 34, 35]. Providing this detail is a noted deficit in implementation science literature, with Proctor and colleagues noting that implementation strategies are inconsistently labeled, poorly justified, and rarely described in detail [36]. Our study adds to the body of implementation science literature as we provide theoretical justification for the implementation strategies used and offer details of how the strategies were operationalized.

### CHG bathing process and documentation compliance

Admittedly, CHG bathing compliance is difficult to measure. We measured CHG bathing compliance not only by auditing EHR documentation, but also by auditing observations of the actual process of CHG bathing. As this study sought to improve *how* CHG bathing was done—per the AHRQ protocol—it was necessary for us to measure the process steps for completing a CHG bath. In addition to documentation audits, Caya and colleague s[18] also completed observation audits, as well as measured CHG usage data, to monitor compliance. They found that out of 28 baths observed, only 57% were fully compliant with the CHG bathing process. Through observations (and in some instances, self-reported measures), our study found significant improvements in the CHG bathing process—meaning that after using educational outreach visits and audit and feedback implementation strategies, nursing staff were more likely to complete CHG baths the correct way per the AHRQ protocol. These improvements were sustained over the following 12 months.

Whereas documentation may not always reflect nursing practice, the majority of previous articles have measured CHG bathing through auditing the EHR [17–19, 34, 35]. Some studies did note an improvement with CHG bathing documentation [17, 18]. Our study did not find a significant improvement in CHG documentation compliance, possibly due to the fact that CHG bathing was already implemented in these units, and staff were habitual in documenting the bath as standard practice. Interestingly, with regard to documentation, the intervention was more beneficial for units with fewer admissions each month and younger NAs. Younger NAs may be newer and more attentive to EHR documentation. Sustainability audits showed that documentation compliance remained high. At one institution, after the initial intervention, a task reminder was built into the EHR which could play into the sustained effects.

Moderating effects of the intervention on bathing compliance were analyzed by including characteristics of the hospital units in the model. For process compliance, we determined that the intervention was more beneficial for smaller units (with fewer beds), those with a shorter length of stay, fewer RN FTEs, and higher RN hours per patient day. Clinically, these findings are understandable; smaller units with fewer beds (and therefore fewer patients) may find that daily CHG bathing is not as onerous of a task; smaller units may also more easily adopt and adapt to change. Further with more RN hours per patient day, even with less RN FTEs, staff may have more time to complete CHG baths. Finally, units with an average lower length of stay may have patient who are more willing to adhere to the daily CHG bathing protocol; conversely, units with a higher average length of stay may find patients are not as comfortable with CHG bathing as they prefer a “real” bath, as CHG cloths can leave a sticky residue.

Temporal effects of the intervention seen in this study have also been reported in previous studies [37]. In particular, the decrease in the effect of the intervention over time has been seen across varying patient populations, settings, and intervention approaches. These findings reiterate the importance of a “booster” session to reinforce the value and patient benefit of the evidence-based intervention over time [37]. In the case of CHG bathing, booster sessions also address educational gaps that may insidiously occur with staff turnover and new-hires.

Our study showed that increase in CAI scores were associated with a decrease in the effect of the intervention on process compliance, yet an associated increase in the effect of the intervention on documentation compliance. The influence of a strong context moderating a negative effect between the interventions and bathing process compliance, but a positive effect on documentation compliance seems paradoxical. Whereas other context tools have been used to explore moderation of an outcome on the intervention, no such study was found using the CAI [38, 39]. Further psychometric testing may be needed to establish the CAI’s validity as a determinant of prospective implementation. It is conceivable that units with lower context may have benefited from the intervention on their bathing process outcome more so than units with higher context. Conversely, units with higher context may have influenced improved documentation compliance more readily than units with lower context. Further exploration of CAI domain sub-scores and factors is indicated to better understand how units may have differed on total scores and what might explain the moderating effects.

### Knowledge/perceptions of CHG bathing surveys

Nursing staffs’ knowledge and perception of CHG bathing also improved after the implementation intervention. Nursing staff were more knowledgeable on CHG bathing facts, and also rated the priority they gave to CHG bathing higher. Other studies have also noted an improvement in knowledge and perceptions after tailored, focused education on CHG bathing [16, 17].

### CLABSI rates

Our study was not powered to find a statistically significant decrease in CLABSI rates since the event is rare; however, the overall trend of CLABSIs decreased over the study. Twelve months after the intervention, booster sessions were completed with CLABSI rates showing a statistically significant reduction (*p* = .009). This may indicate that there is a long-term intervention effect on CLABSI rates through implementing audit and feedback and educational outreach visits to improve CHG bathing process and documentation compliance as well as nursing knowledge and perceptions. Several other studies have found significant decreases in CLABSIs after implementing CHG bathing protocols [9, 10, 17]. It is important to continue to monitor CLABSI rates long term to assess for changes and to monitor sustainability of the interventions. In October and November 2019, immediately following the intervention, a national backorder of the CHG cloths occurred, with both hospitals affected. A substitute CHG cloth was used during this time and could have contributed to the lack of significant reduction in CLABSI rates. As CHG bathing became well integrated into the daily workflow, there were sustained effects of CLABSI reduction at 12 months.

As health systems increasingly aim to embed research into practice and rethink the best delivery of care, implementation science strategies serve as the basis for an evolving “learning health system”; one that enables patients and clinicians to more efficiently and effectively integrate existing evidence into the real-world care. Other examples across healthcare push this paradigm forward. One such example is the NIH Collaboratory [40], which efficiently conducts large, cluster randomized trials leveraging health systems to test strategies for embedding evidence-based care into clinical practice. Like the Collaboratory, this study tested two proven implementation strategies, educational outreach visits and audit and feedback, to show improvements in adoption of AHRQ guideline recommendations for CHG bathing across 14 units in two health systems. As a result, evidence for *how* to effectively implement science is generated and can be evaluated, in addition to existing evidence for *what* to implement to reduce infections.

### Limitations

Whereas this study provides valuable information and adds to the body of implementation science literature, there are several limitations. First, there was a low response rate for the CHG bathing knowledge and perceptions survey, even though electronic reminders were sent out to staff. Additionally, documentation of CHG bathing in the EHR may not always accurately represent nursing practice. Measuring CHG bathing process compliance through observation audits was challenging to obtain. If champions were unable to observe a bath, they received this information via self-report from the staff providing the bath. Self-reported measures may contain bias, yet several studies have noted self-reports to be useful and accurate of actual behavior [41]. Champions reporting process compliance may have been biased and reported higher compliance, as they are invested in their units. Additionally, as shown in Figs. 1 and 2, some clusters saw an increase in compliance scores before starting their active intervention phase. This could be caused by a Hawthorne effect [42], where nurses’ compliance improved simply because they knew their actions were being audited. Whereas we found a significant reduction in CLABSIs and sustained improvements in process and documentation audits 12 months after implementation, this Hawthorne effect may impact sustainability of compliance efforts, as process and documentation audits were only completed for purposes of this implementation science study.

Further, educational outreach visits and audit and feedback strategies are time consuming and resource intensive. Prior to using these strategies, healthcare systems should determine their ability to fully implement them. Lastly, CLABSIs are a relative rare event and we did not have enough sample size to prove the reduction was statistically significant, although it was clinically significant.

## Conclusion

In conclusion, using evidence-based implementation strategies tailored to local determinants can improve compliance with evidence-based practices. Educational outreach visits and audit and feedback have been shown to change clinician’s behaviors by providing feedback on their performance and education tailored to their experiences. Moving forward, booster sessions with educational outreach visits and audit and feedback strategies may need to be scheduled for continued monitoring of the sustainability of this intervention. Further, champions involved in the study and nursing leadership from the units will be invited to participate in focus groups to evaluate the study’s implementation strategies. This feedback will provide valuable information to support use of these implementation strategies.

## Supplementary Information

**Additional file 1: Supplemental Table 1.** Unit Characteristics

## Data Availability

The datasets analyzed during the current study are available from the corresponding author on reasonable request.

## Abbreviations

CHG: Chlorhexidine gluconate
ICU: Intensive care unit
BMT: Bone marrow transplant
CLABSI: Central line associated blood stream infection
AHRQ: Agency for Healthcare Research and Quality
RN: Registered nurse
NA: Nursing assistant
NHSN: National Healthcare Safety Network
EHR: Electronic health record
FTE: Full time employee
LOS: Length of stay
GLMM: Generalized linear mixed models
CAI: Context Assessment Index
Ppd: Per patient day
ACT: Alberta Context Assessment

## References

[1] Haddadin Y, Regunath H (2019). Central line associated blood stream infections (CLABSI). StatPearls.

[2] Kornbau C, Lee KC, Hughes GD, Firstenberg MS (2015). Central line complications. Int J Crit Illness Injury Sci.

[3] O’Grady NP (2017). Guidelines for the Prevention of Intravascular Catheter-Related Infections (2011).

[4] Pronovost P, Needham D, Berenholtz S, Sinopoli D, Chu H, Cosgrove S, Sexton B, Hyzy R, Welsh R, Roth G, Bander J, Kepros J, Goeschel C (2006). An intervention to decrease catheter-related bloodstream infections in the ICU. New Engl J Med.

[5] Eggimann P, Harbarth S, Constantin M-N, Touveneau S, Chevrolet J-C, Pittet D (2000). Impact of a prevention strategy targeted at vascular-access care on incidence of infections acquired in intensive care. Lancet.

[6] Umscheid CA, Mitchell MD, Doshi JA, Agarwal R, Williams K, Brennan PJ (2011). Estimating the proportion of healthcare-associated infections that are reasonably preventable and the related mortality and costs. Infect Contr Hosp Epidemiol.

[7] O’Neil C, Ball K, Wood H, McMullen K, Kremer P, Jafarzadeh SR, Fraser V, Warren D (2016). A central line care maintenance bundle for the prevention of central line–associated bloodstream infection in non–intensive care unit settings. Infect Contr Hosp Epidemiol.

[8] Popovich KJ, Lyles R, Hayes R, Hota B, Trick W, Weinstein RA, Hayden MK (2012). Relation of chlorhexidine gluconate skin concentration to microbial density on skin of critically ill patients bathed daily with chlorhexidine gluconate. Infect Contr Hosp Epidemiol.

[9] Dicks KV, Lofgren E, Lewis SS, Moehring RW, Sexton DJ, Anderson DJ (2016). A Multicenter pragmatic interrupted time series analysis of chlorhexidine gluconate bathing in community hospital intensive care units. Infect Contr Hosp Epidemiol.

[10] Huang SS, Septimus E, Kleinman K, Moody J, Hickok J, Heim L, Gombosev A, Avery TR, Haffenreffer K, Shimelman L, Hayden MK, Weinstein RA, Spencer-Smith C, Kaganov RE, Murphy MV, Forehand T, Lankiewicz J, Coady MH, Portillo L, Sarup-Patel J, Jernigan JA, Perlin JB, Platt R, ABATE Infection trial team (2019). Chlorhexidine versus routine bathing to prevent multidrug-resistant organisms and all-cause bloodstream infections in general medical and surgical units (ABATE Infection trial): a cluster-randomised trial. Lancet.

[11] Huang SS, Septimus E, Kleinman K, Moody J, Hickok J, Avery TR, Lankiewicz J, Gombosev A, Terpstra L, Hartford F, Hayden MK, Jernigan JA, Weinstein RA, Fraser VJ, Haffenreffer K, Cui E, Kaganov RE, Lolans K, Perlin JB, Platt R (2013). Targeted versus universal decolonization to prevent ICU infection. N Engl J Med.

[12] Agency for Healthcare Research and Quality (2013). Universal ICU decolonization: an enhanced protocol.

[13] Musuuza JS, Guru PK, O’Horo JC, Bongiorno CM, Korobkin MA, Gangnon RE, Safdar N (2019). The impact of chlorhexidine bathing on hospital-acquired bloodstream infections: a systematic review and meta-analysis. BMC Infect Dis.

[14] Climo MW, Yokoe DS, Warren DK, Perl TM, Bolon M, Herwaldt LA, Weinstein RA, Sepkowitz KA, Jernigan JA, Sanogo K, Wong ES (2013). Effect of daily chlorhexidine bathing on hospital-acquired infection. N Engl J Med.

[15] Popovich KJ (2017). Another look at CHG bathing in a surgical intensive care unit. Ann Transl Med.

[16] Hines AG, Nuss S, Rupp ME, Lyden E, Tyner K, Hewlett A (2015). Chlorhexidine bathing of hospitalized patients: beliefs and practices of nurses and patient care technicians, and potential barriers to compliance. Infect Contr Hosp Epidemiol.

[17] Reynolds SS, Sova C, McNalty B, Lambert S, Granger B (2019). Implementation strategies to improve evidence-based bathing practices in a neuro ICU. J Nurs Care Qual.

[18] Caya T, Musuuza J, Yanke E, Schmitz M, Anderson B, Carayon P, Safdar N (2015). Using a systems engineering initiative for patient safety to evaluate a hospital-wide daily chlorhexidine bathing intervention. J Nurs Care Qual.

[19] Kettelhut V, Van Schooneveld T, McClay J, Fruhling A, Dempsey K (2017). The utility of electronic health record-based hygiene notes for chlorhexidine bathing practice evaluation. J Infect Prevent.

[20] Reagan KA, Chan DM, Vanhoozer G, Stevens MP, Doll M, Godbout EJ, Cooper K, Pryor RJ, Hemphill RR, Bearman G (2019). You get back what you give: Decreased hospital infections with improvement in CHG bathing, a mathematical modeling and cost analysis. Am J Infect Contr.

[21] Institute of Medicine (US) Committee on Quality of Health Care in America (2001). Crossing the Quality Chasm: A New Health System for the 21st Century.

[22] Chan WV, Pearson TA, Bennett GC, Cushman WC, Gaziano TA, Gorman PN, et al. ACC/AHA Special Report: Clinical Practice Guideline Implementation Strategies: a summary of systematic reviews by the NHLBI implementation science work group: A Report of the American College of Cardiology/American Heart Association Task Force on Clinical Practice Guidelines. Circulation 2017;135(9). [cited 2020 Mar 31]. Available from: 10.1161/CIR.0000000000000481 10.1161/CIR.0000000000000481 28126839

[23] Septimus E, Hickok J, Moody J, Kleinman K, Avery TR, Huang SS, Platt R, Perlin J (2016). Closing the translation gap: toolkit-based implementation of universal decolonization in adult intensive care units reduces central line–associated bloodstream infections in 95 community hospitals. Clin Infect Dis.

[24] Musuuza JS, Sethi AK, Roberts TJ, Safdar N (2017). Implementation of daily chlorhexidine bathing to reduce colonization by multidrug-resistant organisms in a critical care unit. Am J Infect Contr.

[25] Steneck NH. Introduction to the Responsible Conduct of Research. American Psychological Association; 2007 [cited 2021 Mar 20]. Available from: 10.1037/e638422011-001

[26] Grol R, Wensing M, Eccles M, Davis D (2013). Improving patient care: the implementation of change in health care.

[27] O’Brien MA, Rogers S, Jamtvedt G, Oxman AD, Odgaard-Jensen J, Kristoffersen DT, et al. Educational outreach visits: effects on professional practice and health care outcomes. Cochrane Database Syst Rev 2007;4. [cited 2020 Mar 31]. Available from: 10.1002/14651858.CD000409.pub2/full 10.1002/14651858.CD000409.pub2 PMC7032679 17943742

[28] Ivers N, Jamtvedt G, Flottorp S, Young JM, Odgaard-Jensen J, French SD, et al. Audit and feedback: effects on professional practice and healthcare outcomes. Cochrane Database Syst Rev 2012;6. [cited 2020 Mar 31]. Available from: 10.1002/14651858.CD000259.pub3/full 10.1002/14651858.CD000259.pub3 PMC11338587 22696318

[29] Rycroft-Malone J, Kitson A, Harvey G, McCormack B, Seers K, Titchen A, Estabrooks C (2002). Ingredients for change: revisiting a conceptual framework. BMJ Qual Safe.

[30] McCormack B, McCarthy G, Wright J, Coffey A (2009). Development and testing of the context assessment index (CAI). Worldviews Evid Based Nurs.

[31] National Healthcare Safety Network (NHSN) (2019). ACH Surveillance for BSI (CLABSI).

[32] Hussey MA, Hughes JP (2007). Design and analysis of stepped wedge cluster randomized trials. Contemp Clin Trials.

[33] Hemming K, Haines TP, Chilton PJ, Girling AJ, Lilford RJ (2015). The stepped wedge cluster randomised trial: rationale, design, analysis, and reporting. BMJ.

[34] Jusino-Leon GN, Matheson L, Forsythe L (2019). Chlorhexidine gluconate baths: supporting daily use to reduce central line–associated bloodstream infections affecting immunocompromised patients. Clin J Oncol Nurs.

[35] Medina A, Serratt T, Pelter M, Brancamp T (2014). Decreasing central line–associated bloodstream infections in the non-ICU population. J Nurs Care Qual.

[36] Proctor EK, Powell BJ, McMillen JC (2013). Implementation strategies: recommendations for specifying and reporting. Implement Sci.

[37] Hefner JL, Tripathi RS, Abel EE, Farneman M, Galloway J, Moffatt-Bruce SD (2016). Quality improvement intervention to decrease prolonged mechanical ventilation after coronary artery bypass surgery. Am J Crit Care.

[38] Yamada J, Squires JE, Estabrooks CA, Victor C, Stevens B. The role of organizational context in moderating the effect of research use on pain outcomes in hospitalized children: a cross sectional study. BMC Health Serv Res. 2017;17 [cited 2020 July 14]. Available from: https://www.ncbi.nlm.nih.gov/pmc/articles/PMC5259896/. 10.1186/s12913-017-2029-2 PMC5259896 28114940

[39] Förberg U, Unbeck M, Wallin L, Johansson E, Petzold M, Ygge B-M (2016). Effects of computer reminders on complications of peripheral venous catheters and nurses’ adherence to a guideline in paediatric care--a cluster randomised study. Implement Sci.

[40] Weinfurt KP, Hernandez AF, Coronado GD, DeBar LL, Dember LM, Green BB (2017). Pragmatic clinical trials embedded in healthcare systems: generalizable lessons from the NIH Collaboratory. BMC Med Res Methodol.

[41] Eccles MP, Hrisos S, Francis J, Kaner EF, Dickinson HO, Beyer F, Johnston M (2006). Do self- reported intentions predict clinicians’ behaviour: a systematic review. Implement Sci.

[42] Goodwin MA, Stange KC, Zyzanski SJ, Crabtree BF, Borawski EA, Flocke SA (2017). The Hawthorne effect in direct observation research with physicians and patients. J Eval Clin Pract.

