# Supplementary material for: Results of the CHlorhexidine Gluconate Bathing implementation intervention to improve evidence-based nursing practices for prevention of central line associated bloodstream infections Study (CHanGing BathS): a stepped wedge cluster randomized trial
Source: Implement Sci. 2021 Apr 26;16:45. doi: 10.1186/s13012-021-01112-4 (PMC8074470; doi:10.1186/s13012-021-01112-4)
Supplement: Supplementary file 1 — Additional file 1: Supplemental Table 1. Unit Characteristics [file 13012_2021_1112_MOESM1_ESM.docx]

Results of the **CH**lorhexidine **G**luconate **Bath**ing Implementation Intervention to Improve Evidence-Based Nursing Practices for Prevention of Central Line Associated Bloodstream Infections **S**tudy (**CHanGing BathS)**: A Stepped Wedge Cluster Randomized Trial

**Supplemental Table 1.** Unit Characteristics

|  | **AHC SICU^A^** | **AHC CICU^A^** | **CH CVICU^A^** | **AHC Onc^B^** | **AHC PCICU^B^** | **CH MICU^B^** | **CH PICU^B^** | **AHC CTICU^C^** | **AHC PICU^C^** | **CH STICU^C^** | **CH NCCU^C^** | **AHC PBMT^D^** | **AHC NCCU^D^** | **CH CICU^D^** |
| --- | --- | --- | --- | --- | --- | --- | --- | --- | --- | --- | --- | --- | --- | --- |
| # of beds | 24 | 17 | 20 | 31 | 17 | 9 | 10 | 32 | 16 | 9 | 8 | 16 | 24 | 18 |
| CL utilization | 1.61 | 1.61 | 5.01 | 1.24 | 1.37 | 2.96 | 9.05 | 1.05 | 2.40 | 3.04 | 3.96 | 1.15 | 3.65 | 2.78 |
| RN hours ppd | 22.09 | 21.95 | 20.87 | 10.25 | 27.03 | 20.87 | 21.66 | 26.24 | 27.91 | 20.08 | 20.87 | 18.51 | 21.4 | 19.7 |
| # RN FTE | 76.87 | 76.54 | 29.2 | 61.24 | 77.89 | 29.21 | 22.24 | 155.62 | 49.96 | 27.39 | 27.39 | 31.08 | 94.69 | 41.83 |
| Staff Turnover | 28.31% | 39.63% | 16.7% | 0.00% | 23.03% | 5.7% | 20% | 27.45% | 30.84% | 6% | 17.8% | 25.1% | 14.6% | 18% |
| Skill Mix: RN Nursing care hours as a % of all nursing care hours | 93.7% | 97.4% | 86% | 78.2% | 95.3% | 86% | 83% | 97.8% | 88.9% | 86% | 88% | 89.2% | 93% | 82% |
| # of admissions per month | 135 | 121 | 167 | 98 | 51 | 83 | 79 | 176 | 91 | 84 | 73 | 22 | 157 | 167 |
| LOS | 4.41 | 4.09 | 2.52 | 11.69 | 8.93 | 2.88 | 1.99 | 4.29 | 3.57 | 2.74 | 3.03 | 14.75 | 4.22 | 2.52 |
| Total years of RN experience | 7.23 | 4.26 | 10.57 | 9.42 | 5.58 | 14.04 | 10.02 | 4.51 | 5.46 | 8.63 | 8.70 | 7.74 | 4.88 | 15.15 |
| Total years of NA experience | 4 | 5 | 15.40 | 8.67 | 3.17 | 12.54 | 6.96 | 8 | 4.4 | 16.00 | 13.96 | 5.8 | 5.86 | 15.44 |
| Average RN age | 30.99 | 28.26 | 37.58 | 36.38 | 29.19 | 40.53 | 33.78 | 28.84 | 29.75 | 34.58 | 35 | 32.25 | 29.39 | 42 |
| Average NA age | 33.31 | 41.86 | 43.67 | 33.20 | 27.19 | 38 | 35.2 | 35.96 | 27.38 | 44 | 33.5 | 31.18 | 39.08 | 48 |

A=Group randomized in June; B=Group randomized in July; C=Group randomized in August; D=Group randomized in September

CL=Central Line; ppd=per patient day; FTE=Full Time Employee; LOS=Length of Stay; CH=Community Hospital; AHC=Academic Health Center; ICU=Intensive Care Unit; SICU=Surgical ICU; CICU=Cardiac ICU; CVICU=Cardiovascular ICU; Onc=Oncology; PCICU=Pediatric Cardiac ICU; MICU=Medical ICU; PICU=Pediatric ICU; CTICU=Cardiothoracic ICU; STICU=Surgical Trauma ICU; NCCU=Neuro Critical Care Unit; PBMT=Pediatric Bone Marrow Transplant Unit
